# Stress-driven fluid flow controls long-term megathrust strength and deep accretionary dynamics

**DOI:** 10.1038/s41598-019-46191-y

**Published:** 2019-07-04

**Authors:** Armel Menant, Samuel Angiboust, Taras Gerya

**Affiliations:** 1 Université de Paris, Institut de physique du globe de Paris, CNRS, F-75005 Paris, France; 2 Present Address: GFZ Helmholtz Centre Potsdam, German Research Centre for Geosciences, Telegrafenberg, 14473 Potsdam Germany; 3 Institute of Geophysics, Swiss Federal Institute of Technology (ETH), Zürich, Switzerland

**Keywords:** Geodynamics, Tectonics

## Abstract

The heterogeneity of frictional strength along the megathrust earthquake zone critically controls plate coupling and long-term subduction dynamics. However, the persistence and distribution of high-friction segments through space and time remain poorly constrained. Here, we show that accretion processes, such as tectonic underplating (i.e., basal accretion of material below the fore-arc region), can be used as a proxy to characterize the long-term frictional zonation of the subduction interface. We carry out numerical thermo-mechanical experiments, which predict a first-order control of tectonic-stress variations on fluid transport in deep fore-arc regions. Accordingly, positive feedback between fluid distribution and effective stress favours the stability of the interface frictional properties at Myr-scale which, in turn, controls the deep accretionary dynamics. We propose that the recognition of thick duplex structures resulting from successive underplating events over tens of Myr, allows for tracking subduction segments exhibiting an increasing frictional behaviour. Our numerical results help ascertain the long-term hydro-mechanical properties and distribution of coupling/decoupling segments of megathrust earthquake zones worldwide where active tectonic underplating is recognized.

## Introduction

Subduction fluids are intricately linked to tectonic processes^1,2^ and have paramount effects on the frictional strength of subduction interfaces and, therefore, on the seismogenic behaviour of megathrust faults^3–5^. Based on seismological and geodetic observations, interfaces appear highly segmented with a heterogeneous distribution of coupled and decoupled patches due to pore fluid pressure variations^6–8^, but also geological and structural complexities of the lower and upper plates^9^. However, the persistence of this short-term frictional pattern is difficult to confirm despite crucial implications at various timescales, such as predicting potential ruptures on the interface^10^ and assessing margin dynamics over thousands to millions of years^11,12^.

The accretion of downgoing material at the base of the fore-arc region^13^ (i.e., tectonic underplating) has been proposed to be triggered by local variations of plate coupling^14^ and may, to this end, shed light on hydro-mechanical processes and stress distribution along the plate interface. However, the exact connection between interface properties, deformation mechanisms and detachments of tectonic slices remains elusive and requires to deal with pore fluid pressure and effective stress interrelations. In this context, we assess for the first time the relations between long-term fluid flow, stress regime and tectonic underplating events in deep fore-arc regions, using high-resolution, two-dimensional, thermo-mechanical experiments.

## Modelling Tectonic Underplating and Fluid Transport

In this study, we reproduce a self-consistent ocean-continent subduction zone governed by conservation laws and visco-elasto-plastic rheologies^15^, solved in a high-resolution domain (i.e., 0.5 and 0.4 km between each node in *x* and *y* directions, respectively). The model takes into account hydration and dehydration processes by implementing compaction and thermodynamically constrained metamorphic reactions^16^, as well as fluid transport driven by fluid buoyancy and dynamic pressure gradients^11,17^. As a result, fluids defined as Lagrangian markers move from high- to low-stress regions, depending on calculated deviatoric stresses (see *Methods* and Supplementary Fig. S1 for details on the modelling procedure and the initial setup).

### Modelling strategy

To assess the variability of subduction zones worldwide, we performed a set of numerical experiments by varying the cooling age of the subducting oceanic lithosphere (i.e., 53 Myr and 20 Myr), the plate convergence rate (i.e., from 4 to 8 cm yr^−1^) and the overriding continental crust thickness (i.e., 30 and 40 km thick; Supplementary Table S1). Aside from these parameters, the main uncertainty on fluid transport calculation is the reference percolation velocity (*v*_*perc*_; see *Methods*). In our reference model, *v*_*perc*_ = 1 cm yr^−1^, which is of same order of magnitude than estimations for long-term fluid flow in accretionary wedges^18,19^. However, these estimations have been obtained from *in-situ* measurements and numerical modelling on the most frontal part of the accretionary wedge and the uncertainty on the fluid velocity remains high in the deep fore-arc region. Accordingly, additional experiments have been carried out by varying *v*_*perc*_ to explore the effect of fluid drainage on plate interface strength and accretion processes (Supplementary Table S1).

### Results from the cold subduction model

**Figure 1:**
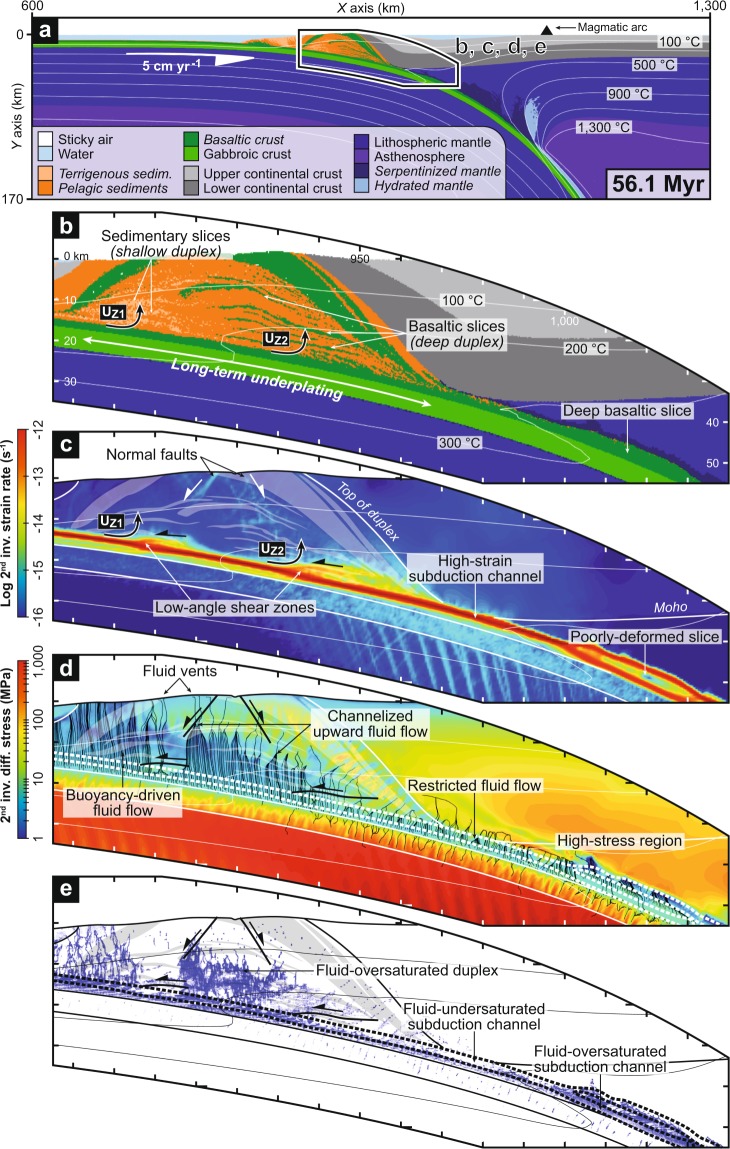
Cold subduction zone and fluid flow pattern (reference model *cold30-5-1*). (**a**) Compositional map. Overview of the subduction zone. Rock types with initially-prescribed pore water content are italicized. (**b**) Compositional map. Zoom on the deep fore-arc region. (**c**) Strain-rate map. (**d**) Differential-stress map. The black streamlines indicate fluid flow pattern calculated according to dynamic pressure gradients. (**e**) Fluid-distribution map. Fluid markers (in blue) indicate local fluid oversaturation. See Fig. 2a for details on the identification of the preferential loci for underplating *U*_*Z1*_ and *U*_*Z2*_. Thick dashed lines (in white and black on panels **d** and **e**, respectively) depict the subduction channel.

**Figure 2:**
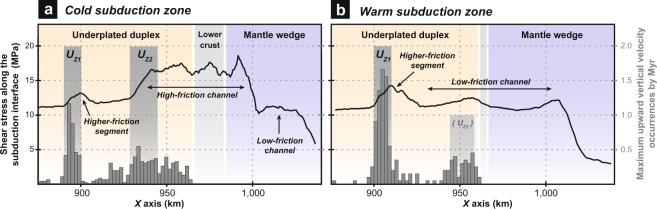
Relations between shear-stress variations along the subduction channel and discrete underplating events in (**a**) cold and (**b**) warm subduction models. Line charts show the shear-stress evolution within the subduction channel integrated over a 1-Myr-long period (i.e., 55-56 Myr and 43–44 Myr for the cold and warm subduction models, respectively). Bar charts show the horizontal distribution of the maximum upward vertical velocity component at the top of the subduction channel for each time step, which is thought to reflect the distribution of underplating events along the plate interface. The main underplating loci *U*_*Z1*_ and *U*_*Z2*_ are highlighted by grey bands. In the warm subduction model, the underplating locus *U*_*Z2*_ is only active until ~35 Myr (see Supplementary Fig. S8 for details).

The reference experiment *cold30-5-1* reproduces the subduction of an old and cold oceanic lithosphere below a 30-km-thick continental lithosphere at a convergence rate of 5 cm yr^−1^. Accretion dynamics of this long-lived subduction zone is dominated by a succession of tectonic underplating events of sedimentary and basaltic material inserted at the base of the fore-arc crust as discrete tectonic slices (Fig. 1 and Supplementary Movie S1). This leads to the formation of an ~80-km-wide dome-shaped structure, so-called duplex, after ~56 Myr of convergence. The most superficial terrigenous sedimentary layer of the subducting plate is underplated at ~16–17 km depth, whereas basaltic slices detach preferentially at ~24–25 km depth, forming two sub-duplex structures (i.e., underplating loci *U*_*Z1*_ and *U*_*Z2*_, respectively; Fig. 1b). Interestingly, these underplating loci localize in the frontal part of persistent low-angle compressional shear zones rooting into the subduction channel (Fig. 1c). After each underplating event, the main active shear zone is successively stepping down and previously accreted slices are passively exhumed through the fore-arc crust. We stress that the along-dip positions of *U*_*Z1*_ and *U*_*Z2*_ remain remarkably stable over the entire duration of the experiment (see bar chart in Fig. 2a and Supplementary Fig. S2). At near-surface conditions, high-angle normal faults and erosion control the final exhumation and the unroofing of the duplex (Fig. 1c). Deeper down, the nappe pile gets narrower due to the stiff lower fore-arc crust. At mantle depth, restricted serpentinization atop the subduction channel and weakening of the dehydrating basaltic crust are responsible for the transient formation of a thick basaltic slice, which is not accreted to the overriding plate due to the persistence of the weak serpentinite layer (Fig. 1b,c).

As shown in Fig. 1d,e, fluid markers released from the subducting sediments and upper basaltic crust mostly migrate vertically upward throughout the duplex, creating fluid-oversaturated conditions. This transport is driven by fluid buoyancy, which prevails over low deviatoric stresses associated with passive exhumation of the duplex. In the shallow parts of the duplex, high-angle normal faults locally generate a low-stress region (i.e., tectonic underpressure) channelizing fluids until they reach the surface as localized vents (Fig. 1d). Tectonic underpressure also drives fluids downward throughout the gabbroic crust and underlying lithospheric mantle through tensile fractures related to slab bending^17^. In contrast, local tectonic overpressure patches controlled by low-angle shear zones hamper upward fluid transport atop the plate interface (Fig. 1d). Note that our model does not predict significant updip (or downdip) fluid flow along the plate interface. Near the tip of the mantle wedge corner, high compressional stresses in the fore-arc lower crust are responsible for a relative scarcity of free fluids and the transmission of shear stress to the subduction channel (Figs 1e and 2a). High stresses (up to ~300 MPa) extend deeper down into the subcontinental mantle, restricting upward fluid flow from the plate interface and, consequently, the serpentinization degree of the mantle wedge over the entire duration of the experiment (Fig. 1d). The crossing of the 300 °C isotherm activates thermodynamically-constrained metamorphic reactions^11,16^, resulting in fluid oversaturation and a significant decrease of the depth-integrated shear stress of the subduction channel at mantle depth (Figs 1e, 2a and Supplementary Fig. S2).

**Figure 3:**
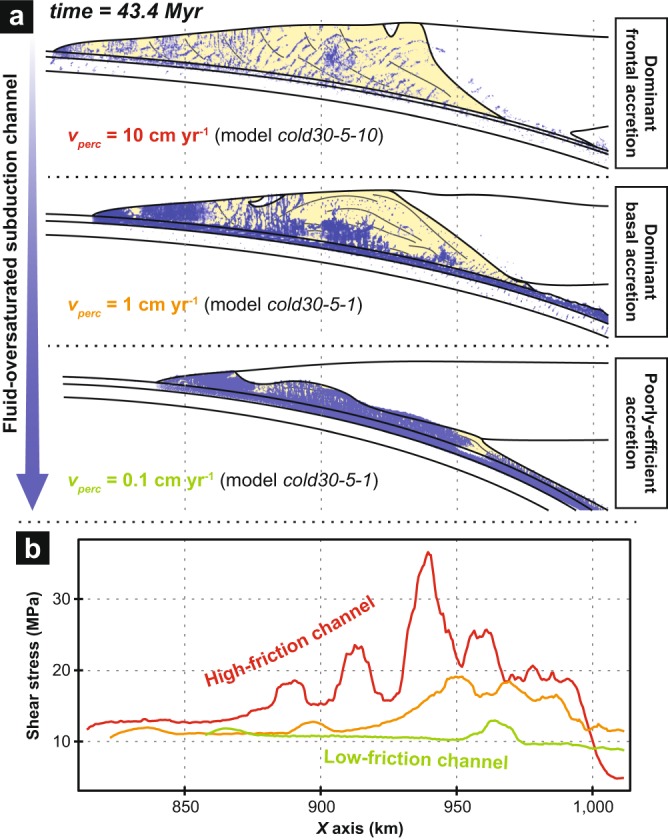
Correlation between fluid distribution, frictional properties of the plate interface and fore-arc accretion dynamics. (**a**) Model-based sketches show the first-order geometry of the fore-arc margin for three simulations displaying different reference percolation velocity (*v*_*perc*_). Accreted material is in yellow. Fluid markers (in blue) indicate local fluid oversaturation. See Fig. 1 and Supplementary Figs S6 and S7 for details on models *cold30-5-1*, *cold30-5-0*.*1* and *cold30-5-10*, respectively. (**b**) Line chart shows shear-stress variations within the subduction channel integrated over 1-Myr-long. Green, orange and red lines correspond to models with *v*_*perc*_ equal to 0.1, 1 and 10 cm yr^−1^, respectively.

Fluid transport and the overall margin dynamics are consistently reproduced in additional experiments with a variable plate convergence rate (i.e., models *cold30-4-1* and *cold30-8-1*; Supplementary Figs S3 and S4) and thicker overriding crust (i.e., model *cold40-5-1*; Supplementary Fig. S5), suggesting that these results are robust for a wide range of first-order parameters characterising active margins worldwide. Alternatively, variations in fluid transport properties (i.e., *v*_*perc*_) result in significantly different fluid distribution and accretion dynamics (Fig. 3). Thus, a low *v*_*perc*_ (i.e., model *cold30-5-0*.*1*; Supplementary Fig. S6) restricts fluid flow and promotes fluid-oversaturated conditions along the plate interface and immediately above, resulting in a low-friction channel and a poorly efficient fore-arc accretion. Alternatively, increasing *v*_*perc*_ leads to a higher-friction channel and more efficient basal then frontal accretion processes (i.e., reference model *cold30-5-1* and model *cold30-5-10*, respectively; Fig. 3 and Supplementary Fig. S7). For details on the results from these additional experiments, the reader is referred to Supplementary Text.

### Modulation of fluid transport and accretion dynamics in the warm subduction model

**Figure 4:**
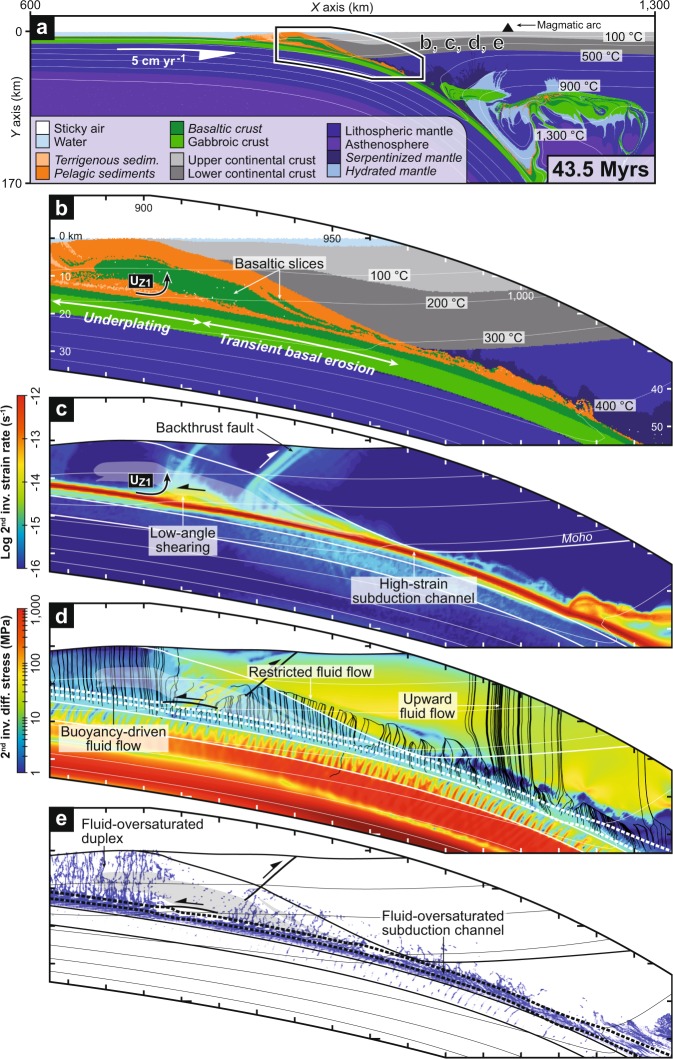
Warm subduction zone and fluid flow pattern (model *warm30-5-1*). (**a**) Compositional map. Overview of the subduction zone. Rock types with initially-prescribed pore water content are italicized. (**b**) Compositional map. Zoom on the deep fore-arc region. (**c**) Strain-rate map. (**d**) Differential-stress map. The black streamlines indicate fluid flow pattern calculated according to dynamic pressure gradients. (**e**) Fluid-distribution map. Fluid markers (in blue) indicate local fluid oversaturation. See Fig. 2b for details on the identification of the preferential locus for underplating *U*_*Z1*_. Thick dashed lines (in white and black on panels **d** and **e**, respectively) depict the subduction channel.

Modelling the subduction of a younger and warmer oceanic lithosphere (i.e., model *warm30-5-1*) results in a similar evolution as for the cold subduction model during the first ~20 Myr, with the formation of an early duplex by successive underplating events (Fig. 4 and Supplementary Movie S2). Latter in the model evolution, the predominance of basal erosion over tectonic underplating along the plate interface at ~20–30 km depth leads the partial consumption of the accreted structure, which is only maintained by persistent underplating at ~15–16 km depth (underplating locus *U*_*Z1*_; Figs 2b, 4b and Supplementary Fig. S8). On top of this small duplex, the fore-arc margin is affected by a long-term subsidence and forward and backward thrusting (Fig. 4b,c). Despite such discrepancies in margin dynamics, the fluid circulation pattern in the warm subduction model is relatively consistent with the cold subduction setting (Fig. 4d,e), except that (i) thermodynamically-constrained release of slab-derived fluids occurs at shallower depth (i.e., ~25–30 km depth), resulting in a low-friction, fluid-oversaturated subduction channel near the base of the fore-arc crust (Fig. 2b) and (ii) thermal weakening of the mantle wedge allows for lower stress accumulation and vertical fluid migration up to the continental crust (Fig. 4d).

## Discussion

### High stress and fluid trapping

All our numerical experiments evidence the formation of a more or less developed duplex by accretion of sedimentary and basaltic slices at the base of the fore-arc crust, independently from the age of the oceanic lithosphere, the plate convergence rate and the thickness of the overriding crust (Figs 1, 4 and Supplementary Figs S3, S4 and S5). Similar nappe stacking has been largely recognized from paleo-accretionary complexes, including the Franciscan complex (Western US)^20^ and the Western Series (Central-Southern Chile)^21,22^. Geophysical imaging of duplex structures has also been reported from several actives subduction zones^23–26^ (e.g., Alaska, central Japan, Cascadia, New Zealand), leading to suggest that tectonic underplating may be ubiquitous along most of active margins worldwide.

**Figure 5:**
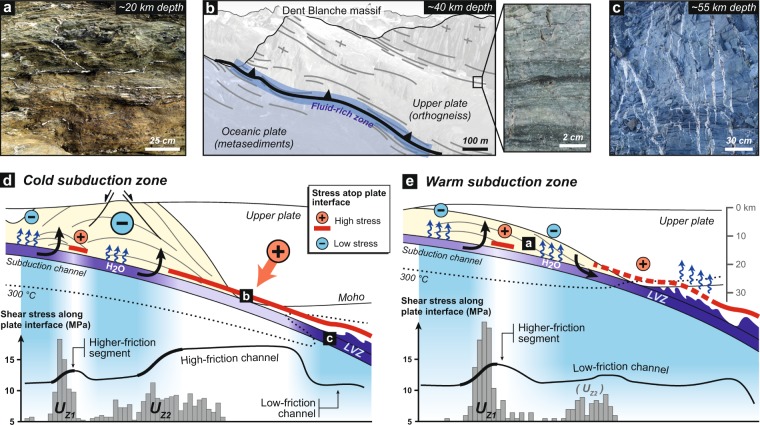
Fluid distribution, hydro-mechanical properties of the plate interface and preferential location of tectonic underplating in cold and warm subduction settings. Field evidences for fluid distributions at various depths from ancient subduction zones allow for supporting model results (see location of panels a,b,c on panels d and e). (**a**) Foliation-parallel and nearly vertical quartz veins crosscutting greenschist-facies metasediments (Western Series, Central Chile, high thermal gradient). (**b**) Interpreted panoramic view of the Dent Blanche basal tectonic contact (Western Alps, intermediate thermal gradient). Foliated orthogneiss located several hundreds of meters above the interface displays no evidence of fluid percolation (see inset). (**c**) Hydrofractured blueschist-facies metavolcanics (Zagros, Iran, low thermal gradient). (**d**,**e**) Model-based sketches linking hydro-mechanical properties of the plate interface with tectonic underplating events in cold and warm subduction zones, respectively. Line charts show shear-stress variations along the interface. Bar charts show the horizontal distribution of underplating events along the plate interface for the entire model duration (see Fig. 2 for details on these charts). Blue bands depict the relative proportion of fluid markers (i.e., fluid-oversaturated conditions) into the subduction channel. Blue arrows depict the dominant fluid flow pattern. LVZ: Low-velocity zone.

Predicted fluid distribution in the cold and warm subduction models (Figs 1e, 4e and 5) is globally consistent and supported by numerous field and seismological observations from ancient and active subduction zones, which provide robust evidences of pore fluid pressures near lithostatic values along the plate interface^27,28^. These include hydrofracture networks into fossilized subduction channels from 10 to >40 km depth^29,30^, as well as seismic imaging of a 2–8-km-thick, landward-dipping low-velocity zone (LVZ) with high *V*_*P*_/*V*_*S*_ ratio near the mantle wedge corner along several active margins displaying contrasting thermal regimes^27^ (e.g., SW Japan, Cascadia, Alaska). It is noticeable that fluid release from thermodynamically-constrained metamorphic reactions are activated at shallower depth (i.e., ~25–30 km depth) in the warm subduction model, resulting in a fluid-oversaturated subduction channel at the base of the fore-arc crust (Figs 4e and 5e), which is in line with the shallow LVZs monitored along warm active margins^31,32^ (e.g., Cascadia, SW Japan). Independently from the thermal regime, these high fluid pressures must be achieved by undrained, low-permeability conditions at the top of the plate interface^31,33–35^. Several hypotheses to explain this phenomenon have been proposed such as impermeable lithologies^36^, deformation-controlled grain-size reduction processes^37^ or mineral precipitation^38^. According to our numerical experiments, we argue that tectonic-stress variations and associated dynamic pressure gradient on and above the plate interface exert a first-order control on fluid transport (Figs 1d, 4d and 5). Thus, the passively-exhuming duplex displays low deviatoric stresses, which allow for buoyancy-driven vertical fluid migration, in line with field observations worldwide showing abundant, nearly vertical, vein systems emplaced during the exhumation of paleo-accretionary duplexes^39–41^ (Fig. 5a). Deeper down, the high differential stresses in the lower fore-arc crust of the cold subduction model partly close the channel at 30–35 km depth (in line with the conceptual vision of Cloos and Shreve^42^) and avoid extensive fluid influx above the interface (Fig. 1d). The Dent Blanche massif (NW Alps) exposes a natural analogue corresponding to this situation, which has been subsequently exhumed during the Alpine orogeny^43^ (Fig. 5b). Here, a strongly-hydrated 100–200-m-thick fossilized subduction interface is overlain by plastically-deformed orthogneiss almost devoid of evidence for hydrofracturing (see inset in Fig. 5b). Higher up in the Dent Blanche massif, granulite-facies rocks show numerous pseudotachylyte-bearing faults formed at ~30 km depth under differential stresses of several hundreds of MPa^44^, in agreement with estimates yield by our numerical results (Fig. 1d). At 35–55 km depth, the cold subduction model predicts a 2–4-km-thick fluid-oversaturated layer corresponding to the basaltic crust and the overlying channel (Fig. 1e). The formation of this fluid-rich zone is the consequence of persisting high stresses in the subcontinental mantle and massive fluid release via metamorphic reactions, which is supported by seismic imaging of the LVZs worldwide^27^ and by field evidences of intense veining along the plate interface at similar depths^30,45^ (Fig. 5c).

The lack of updip fluid flow along the subduction channel in our experiments apparently departs from numerical, experimental and field-based observations suggesting interface-parallel migration^43,46,47^. The discrepancy can be explained by the calculation of fluid transport in our models, which does not consider any porosity and permeability variations that are thought to be anisotropic and to occur at multiple spatial and temporal scales^46,48^. Indeed, a high-permeability subduction channel would favour along-dip fluid flow with respect to dynamic pressure gradients. However, it is noteworthy that massive upward fluid percolation from the deep subduction interface is suspected notably in warm subduction settings, based on seismically-imaged low-Poisson’s ratio anomalies into the fore-arc crust^38,49^, as well as on the mantle-derived isotopic signature of mineral springs located at the surface^50,51^. This statement is supported by our modelling results, which predict an upward fluid infiltration through a persistent low-stress, thermally-weakened mantle wedge above a young and warm oceanic lithosphere (Fig. 4d). Alternatively, the maintaining of high pore fluid pressures in the deep fore-arc region is suspected from ancient and active subduction zones^27,28^, indicating important fluid accumulation over long timescales, responsible for partial serpentinization of the mantle wedge^52^. All these sensibly different observations make it difficult to decipher the respective contribution of interface-parallel *versus* across-interface fluid flow at Myr timescales. In view of our set of experiments, we suggest that the magnitude of the along-dip transport may be marginal with respect to the long-term fluid budget.

### Tectonic underplating as a window on plate-interface frictional properties

The aforementioned heterogeneous distribution of fluid-oversaturated zones within the subduction channel in a cold regime results in along-dip shear-stress variations in the range of ~5–18 MPa with two spatially and temporally stable higher-friction regions (Fig. 2a and Supplementary Fig. S2). A clear correlation is observed between these persistent higher-friction zones and the two *U*_*Z1*_ and *U*_*Z2*_ segments where tectonic underplating proceeds. Indeed, underplating appears to be triggered by the transition from low to higher subduction-channel shear stresses (and hence, from a wetter to a drier interface segment; Fig. 5d). This correlation is supported by similar results from models with a variable plate convergence rate (Supplementary Figs S3 and S4), a thicker overriding crust (Supplementary Fig. S5) or a younger and warmer subducting oceanic lithosphere (Figs 2b and 5e). Thus, it seems that the genetic link between subduction segments exhibiting an increasing frictional behaviour and tectonic slicing is robust, independently of the kinematics and the thermal regime of the active margin. However, it is noteworthy that, in the case of a warm subduction zone, the higher-friction zone near *U*_*Z1*_ is temporally stable while the patch near *U*_*Z2*_ progressively disappears (Fig. 2b and Supplementary Fig. S8). Accordingly, margin dynamics is modified as underplating at *U*_*Z1*_ is overthrown by basal erosion at *U*_*Z2*_, leading to the partial consumption of the former duplex structure (Fig. 4 and Supplementary Movie S2). These results support the earlier proposed conceptual model suggesting that high pore fluid pressures along the interface (and associated hydrofracturing) promote long-term basal erosion, as shown by seismic imaging at erosional margins^53,54^ (e.g., Costa Rica, Ecuador). Furthermore, additional experiments show that variations in fluid transport properties strongly control the frictional state of the plate interface and, therefore, the efficiency and the depth of accretion processes (Fig. 3 and Supplementary Figs S6 and S7; see also Supplementary Text). Thus, the more drained (and hence strengthened) the subduction channel is, the more accretive the margin is, because of an increasing coupling between the subducting material and the top of the subduction channel.

The positive feedback existing between stresses and fluids^2^ allows to maintain this correlation pattern between fluid distribution, high-friction subduction segments and deep accretion, both in space and time. Indeed, low-stress domains favour fluid influx which, in turn, enhances rheological weakening and hence stress lowering. Conversely, higher stresses cause fluid escape, which further increases stresses on the plate interface, ultimately triggering local underplating. The long-term consequence of this self-supported mechanism is the growth of accretionary wedges and duplex structures formed over tens of millions of years (Figs 1, 3 and 5d), strikingly recalling those identified in analogue modelling experiments^21,55^, previous numerical studies^56^ and field-based conceptual models^13,22,57^. However, the natural record of very large duplexes such as the one formed after 56 Ma of convergence in our reference model (Fig. 1) are rare, as it is unlikely to maintain a stable strength regime of the interface over such a long period of time. Changes in the rheological properties of the subduction channel (e.g., subduction of topographic highs, variably-hydrated incoming plate) may lead to a switch from an accretionary to an erosional mode and hence to the basal erosion of the previously-formed duplex structure^11,53^.

A major implication from our study is that accretionary processes, and especially tectonic underplating, may be used as a proxy to ascertain the frictional properties of the plate interface over Myr timescales. Only a limited number of geophysical studies tentatively link active tectonic slicing, plate coupling and stress distribution and all of them tackle with a loss of resolution on plate interface processes deeper than 15 km^24,26,58,59^. In addition, GPS-based locking maps show a heterogeneous distribution of coupled subduction segments with a common decrease approaching the subcontinental mantle^8,10^ but the link with long-term underplating is still challenging to establish. Our finding implies that regions where active duplexing is recognized could be viewed, on the short-term point of view, as the consequence of the transition from decoupled to coupled regions along the interface (Fig. 5). In active subduction settings, these patches would not be systematically visible as GPS-monitored frictional heterogeneities, which only reflect transient, short-term processes associated with the seismic cycle.

Last, variations in the frictional properties (and pore fluid pressure) along the plate interface have been invoked to explain slow slip events (SSE) updip and downdip the seismogenic zone^5,60^. Our results suggest that a rheological link between tectonic underplating regions and subduction segments exhibiting SSE must exist. Accordingly, stress must play a fundamental role for controlling fluid distribution and thus, the location of SSE phenomena along the interface.

## Methods

### Governing equations

The two-dimensional numerical experiments are carried out with the *I2ELVIS* code, which solved the continuity, momentum and heat conservation equations, based on a finite difference scheme and a non-diffusive marker-in-cell technique^15^. The continuity equation describes the conservation of mass, assuming a visco-elasto-plastic compressible fluid. It is solved on a staggered Eulerian grid and has the form:

1 $$\frac{D\,\mathrm{ln}\,{\rho }_{eff}}{Dt}+\frac{\partial {v}_{i}}{\partial {x}_{i}}=0$$

where *ρ*_*eff*_ is the effective rock density calculated in Eq. (7), *t* the time, *v*_*i*_ the viscous velocity and *x*_*i*_ the spatial coordinates *x* and *y*. The momentum of the compressible fluid is then solved using the Stokes equation:

2 $$-\frac{\partial P}{\partial {x}_{i}}+\frac{\partial {\sigma }_{ij}}{\partial {x}_{i}}=-\,{\rho }_{eff}\,{g}_{i},$$

where *P* is the pressure, *σ*_*ij*_ the components of the deviatoric stress tensor and *g*_*i*_ the gravitational acceleration (*g*_*x*_ = 0 and *g*_*y*_ = 9.81 m s^−2^). The heat conservation equation is formulated in a Lagrangian form to avoid numerical diffusion of temperature:

3 $${\rho }_{eff}\,{C}_{P}\frac{{\rm{D}}T}{{\rm{D}}t}=-\frac{\partial {q}_{i}}{\partial {x}_{i}}+{H}_{r}+{H}_{a}+{H}_{s},$$

where *C*_*p*_ is the isobaric heat capacity, *T* the temperature, *H*_*r*_ the radiogenic heat production, *H*_*a*_ the adiabatic heat production, *H*_*s*_ the shear heating and *q*_*i*_ the heat flux solved as:

4 $${q}_{i}=-k\frac{\partial T}{\partial {x}_{i}},$$

where *k* is the thermal conductivity depending pressure, temperature and rock type (Supplementary Table S2).

### Fluid implementation

In our experiments, fluids are initially prescribed in the subducting oceanic lithosphere as (i) pore water in sediments and basaltic crust (${X}_{{w}_{pore}}=1$ wt.%) and (ii) mineral bound water in sediments, basaltic crust and gabbroic crust. Pore water release by compaction is assumed constant from 0 to 75 km depth, also mimicking dehydration from low-temperature metamorphic reactions^29^ (e.g., smectite-illite, opal-quartz transformations). This linearly-decreasing pore water content accounts for the kinetics of metamorphic reactions and the natural heterogeneity of rocks that result in distributed fluid release over a temperature range, rather than in discrete pulses^2,61^. Bound water release is calculated by free-energy minimization^16^ as a function of pressure, temperature and rock type^11^. Resulting free water is then transported as newly-formed Lagrangian markers. Importantly, our models do not take into account the coupling between pore pressure and hydraulic properties (i.e., porosity and permeability) as considered in real two-phase flow systems^62^. Instead, we make the following assumptions: (i) fluid pressure approaches the solid dynamic pressure and (ii) hydraulic properties remain constant due to the balance between porosity enhancement and destruction^17^. Accordingly, fluid marker transport is controlled by the Stokes velocity field *v*_*i*_, the fluid buoyancy and the dynamic pressure gradients, such that:

5 $${v}_{{i}_{water}}={v}_{i}+{v}_{perc}\,{k}_{i},$$

where ${v}_{{i}_{water}}$ is the velocity of fluid markers, *v*_*perc*_ the reference percolation velocity (varying from 0.1 to 10 cm yr^−1^ in our experiments; see Supplementary Text and Supplementary Table S1 for details on the parametric study) and *k*_*i*_ the pressure-dependent coefficient calculated as:

6 $${k}_{i}=\frac{{\rho }_{crust}\,{g}_{i}-\frac{\partial P}{\partial {x}_{i}}}{({\rho }_{crust}-{\rho }_{fluid})\,{g}_{y}},$$

where *ρ*_*crust*_ and *ρ*_*fluid*_ are respectively the reference crustal and fluid densities (*ρ*_*crust*_ = 2300 kg m^−3^ and *ρ*_*fluid*_ = 1000 kg m^−3^) and |*k*_*i*_| = 2 for |*k*_*i*_| > 2. Upward transport is defined by positive values. Once moving, fluid markers may be then consumed by rock markers (either as pore or mineral bound water), depending on their stable water content. Further details on the fluid implementation are provided by refs^11,17^.

### Fluid effects on rock density and rheology

In our experiments, bound water content in rock markers affects rock density as follow:

7 $${\rho }_{eff}={\rho }_{rock}(1-{X}_{fluid})+{\rho }_{fluid}\,{X}_{fluid},$$

with

8 $${\rho }_{rock}={\rho }_{0}\,(1-\alpha (T-298))\,(1+\beta \,(P-0.1)),$$

where *ρ*_0solid_ is the standard density of rocks, *X*_*fluid*_ the mass fraction of fluid, *α* the thermal expansion and *β* the compressibility (see Supplementary Table S2 for a complete list of rock properties). Non-Newtonian visco-elasto-plastic rheologies are employed in these experiments, implying that the deviatoric strain rate tensor *ε*_*ij*_ includes the three respective components:

9 $${\dot{\varepsilon }}_{ij}={\dot{\varepsilon }}_{i{j}_{viscous}}+{\dot{\varepsilon }}_{i{j}_{elastic}}+{\dot{\varepsilon }}_{i{j}_{plastic}}.$$

Details on the calculation of the rheological constitutive equations are available in ref.^15^. For our purpose, it is important to note that free fluids affect rock rheology by modifying the plastic strength *σ*_*yield*_, which limits the creeping (i.e., viscous) behaviour such that:

10 $$\eta \le \frac{{\sigma }_{yield}}{2\,{\dot{\varepsilon }}_{II}},$$

with

11 $$\eta ={{\dot{\varepsilon }}_{II}}^{\frac{1-n}{n}}\,{{A}_{D}}^{\frac{1}{n}}\,exp(\frac{E+P\,V}{n\,R\,T}),$$

and

12 $${\sigma }_{yield}=C+P\,\sin ({\phi }_{dry})\,(1-{\lambda }_{fluid})$$

where *η* is the effective creep viscosity, ${\dot{\varepsilon }}_{II}$ the second invariant of the strain rate tensor, *n* the creep exponent, *A*_*D*_ the pre-exponential factor, *E* the activation energy, *V*. the activation volume, *R* the gas constant, *C* the cohesion, *φ*_*dry*_ the internal friction angle for dry rocks and *λ*_*fluid*_ the pore fluid pressure factor. The latter is defined as *λ*_*fluid*_ = 0 for dry rocks and *λ*_*fluid*_ = 0.99 for fluid-oversaturated rocks (indicated locally by the presence of fluid markers) except at the surface where hydrostatic conditions are assumed (i.e., *λ*_*fluid*_ = 0.4). Because no fluid infiltration or alteration is evidenced a few hundreds of meters above fossilized subduction channels^43,63^, fluid weakening effects have been deactivated for the upper and lower continental crusts.

### Numerical setup

In all numerical experiments, the computational domain meases 1500 × 200 km in the *x* and *y* direction, respectively (Fig. S1a). Eulerian grid resolution is 2.0 × 1.5 km except at the centre of the domain where it is 0.5 × 0.4 km. Additionally, ~8 millions of randomly distributed markers are initially prescribed for advecting material properties and computing water release, transport and consumption. Convergence rate (defined within the convergence condition region) is 5 cm yr^−1^. The initial setup is designed with a 30-km-thick overriding continental crust composed of 15 km of felsic upper crust and 15 km of mafic lower crust. The 7.5-km-thick subducting oceanic crust is made up of 0.5 km of pelagic sediments, 2 km of hydrated basaltic crust and 5 km of gabbroic crust (Fig. S1b). The oceanic crust is initially underthrusted below the continental margin and a 10-km-thick weak zone is prescribed at the interface between the two plates to initiate subduction.

The thermal structure of the oceanic lithosphere is initially defined by an oceanic geotherm with a cooling age evolving from 10 kyr (*x* = 0) to 53 Myr (*x* = 854 km) for the cold subduction setting (model *sub53-1*). To limit the size of the computational domain, the cooling of the oceanic lithosphere is prescribed as 10 times faster for 0 ≤ *x* ≤ 200 km. This high cooling zone is located at ~600 km away from the subduction zone, which allows to avoid any thermal or mechanical effect on modelled fore-arc dynamics. In the case of a warm subduction setting (model *sub20-1*), the maximum cooling age of the oceanic lithosphere is 20 Myr and its cooling is only 2 times faster for 0 ≤ *x* ≤ 200 km. A geothermal gradient of ~15° km^−1^ down to 90 km is defined for the continental lithosphere. Below, the asthenospheric geothermal gradient is 0.5° km^−1^.

Velocity boundary conditions are free slip for the left, right and upper boundaries, while the lower boundary is open to ensure mass conservation in the computational domain. The top of the lithosphere is calculated as an internal free surface by using a low-viscosity layer simulating air (*y* < 10 km) or water. Resulting large viscosity contrast minimizes shear stresses at the top of the lithospheres, which efficiently approximates a free surface^64^. In our experiments, sedimentation and erosion processes are implemented independently by applying the following equation at the surface^65^:

13 $$\frac{\partial {y}_{surf}}{\partial t}={v}_{y}-{v}_{x}\frac{\partial {y}_{surf}}{\partial x}-{v}_{sedim}+{v}_{erosion},$$

where *y*_*surf*_ is the *y* coordinate of the surface, *v*_*x*_ and *v*_*y*_ the horizontal and vertical velocity components of the Stokes velocity field at the surface and *v*_*erosion*_ and *v*_*sedim*_ the global erosion and sedimentation rates, respectively, defined as (i) *v*_*erosion*_ = 0.3 mm yr^−1^ and *v*_*sedim*_ = 0 mm yr^−1^ for *y* < 10 km and (ii) *v*_*erosion*_ = *v*_*sedim*_ = 0 mm yr^−1^ for *y* > 10 km. A increased erosion/sedimentation rate of 1 mm yr^−1^ is applied to regions with steep surface slopes (i.e., > 17°) for smoothing the topographic surface. This is particularly relevant for the offshore fore-arc region where the increased sedimentation rate counterbalances the absence of global sedimentation rate prescribed in our experiments. Newly-formed sedimentary rocks are labelled as terrigenous sediments and display the same properties than the pelagic sediments (Supplementary Table S2).

## Supplementary information

Movie S1

Movie S2

Supplementary Information
