# Supplementary material for: Stress-driven fluid flow controls long-term megathrust strength and deep accretionary dynamics
Source: Sci Rep. 2019 Jul 4;9:9714. doi: 10.1038/s41598-019-46191-y (PMC6609719; doi:10.1038/s41598-019-46191-y)
Supplement: Supplementary file 3 — Supplementary Information [file 41598_2019_46191_MOESM3_ESM.pdf]

Stress-driven fluid flow controls long-term megathrust strength and deep accretionary dynamics

Armel Menant[1*], Samuel Angiboust[1], Taras Gerya[2]

[1] *Institut de Physique du Globe de Paris, Sorbonne Paris Cité, Univ. Paris Diderot, CNRS, F-75005 Paris, France*

[2] *Institute of Geophysics, Swiss Federal Institute of Technology (ETH), Zürich, Switzerland*

[*] *Corresponding author: armel.menant@gmail.com*

Supplementary information

Content

- Impact of plate convergence rate and overriding crustal thickness on the frictional properties of plate interface and margin dynamics
- Control of fluid drainage on the frictional properties of plate interface and margin dynamics
- Figures S1 to S8
- Tables S1 and S2
- Supplementary references

Impact of plate convergence rate and overriding crustal thickness on the frictional properties of plate interface and margin dynamics

Subduction zones worldwide display a wide range of plate convergence rate which are thought to have a crucial control on accretion (or erosion) dynamics and associated crustal deformation in the fore-arc and arc regions[66,67]. To assess the importance of this first-order parameter on the hydro-mechanical properties of the subduction channel and accretion processes, two numerical simulations have been carried out by varying plate convergence rate from 4 cm yr^{-1} (model *cold30-4-1*, Fig. S3) to 8 cm yr^{-1} (model *cold30-8-1*, Fig. S4). Furthermore, we tested the potential control of the upper plate properties on accretion dynamics by performing an additional experiment where a thicker (i.e., 40 km thick) overriding continental crust is prescribed (model *cold40-5-1*; Fig. S5).

The three experiments are characterized by a succession of tectonic underplating events, which results in the growth of a ~80-90-km-wide duplex structure composed of a pile of sedimentary and basaltic slices (Figs. S3a, S4a and S5a). These tectonic slices are preferentially inserted to base of the fore-arc crust at ~16-18 and ~23-27 km depth, forming two sub-duplexes, which are particularly well expressed in the fast convergence model (i.e., underplating loci U_{Z1} and U_{Z2}; Figs. S3b, S4b and S5b). Transport and distribution of fluid markers released from the subducting plate is consistent for these two models, with a dominant vertical upward flow throughout the duplex, creating fluid-oversaturated conditions (Figs. S3c, S4c and S5c). In addition, a heterogeneous distribution of fluid markers is predicted within the subduction channel, with notably one or two fluid-undersaturated segments displaying high shear stresses (Figs. S3d, S4d and S5d). Importantly, a spatial correlation is observed between these spatially- and temporally-stable higher-friction zones and U_{Z1} and U_{Z2} where underplating proceeds (Figs. S3d, S4d and S5d). This relation is reproduced in all our experiments (see also Fig. 2)

supporting the existence of a genetic link between subduction segments exhibiting an increasing frictional behaviour and tectonic underplating.

Control of fluid drainage on the frictional properties of plate interface and margin dynamics

The experiment with a low reference percolation velocity (i.e., v_{perc} = 0.1 cm yr^{-1}; model *53-0.1*) is characterized by minor tectonic underplating events, resulting in a ~3-8-km-thick duplex structure formed after ~39 Myr of plate convergence (Figs. S6). In the fore-arc region, the continuous release and slow migration of fluid markers lead the pore fluid pressure to increase along the plate interface and immediately above (Fig. S6c). As a result, the subduction channel displays a low and homogenous shear stress distribution except near the mantle wedge corner where a local higher-friction patch is spatially correlated with the preferential site of underplating U_{Z2} (Fig. S6d). This supports our conclusions on the primary role of increased frictional behaviour of subduction segments on the detachment of tectonic slices. However, as mentioned for warmer subduction settings, the overall predominance of a weak channel prevents the growth of a large duplex structure.

Conversely, by considering a higher reference percolation velocity (i.e., v_{perc} = 10 cm yr^{-1}; model *53-10*), we observe a predominance of frontal accretion process and minor tectonic underplating events, which lead to form a ~130-km-wide accretionary wedge (Figs. S7). In this case, fluid markers migrate fast from the top of the subducting oceanic crust to the overriding fore-arc wedge, resulting in a relatively fluid-depleted, high-friction subduction channel displaying spatially- and temporally-unstable higher-friction patches (Figs. S7c and S7d). Consequently, no preferential locus for triggering tectonic slicing is evidenced, implying that underplating events spread all along the subduction interface from ~10 to ~30 km depth.

Thus, by varying reference percolation velocity, we evidence that deep accretion processes at active margins are favoured if the subduction channel is well drained and displays a high frictional behaviour (Fig. 3). This is in line with the conclusions drawing from analogue modelling experiments, which emphasize the crucial role of high basal friction on tectonic underplating[21,55,68]. However, our numerical results further demonstrate that stress-controlled fluid flow causes significant variations of the frictional properties and changes of the accretion dynamics along-dip the plate interface.

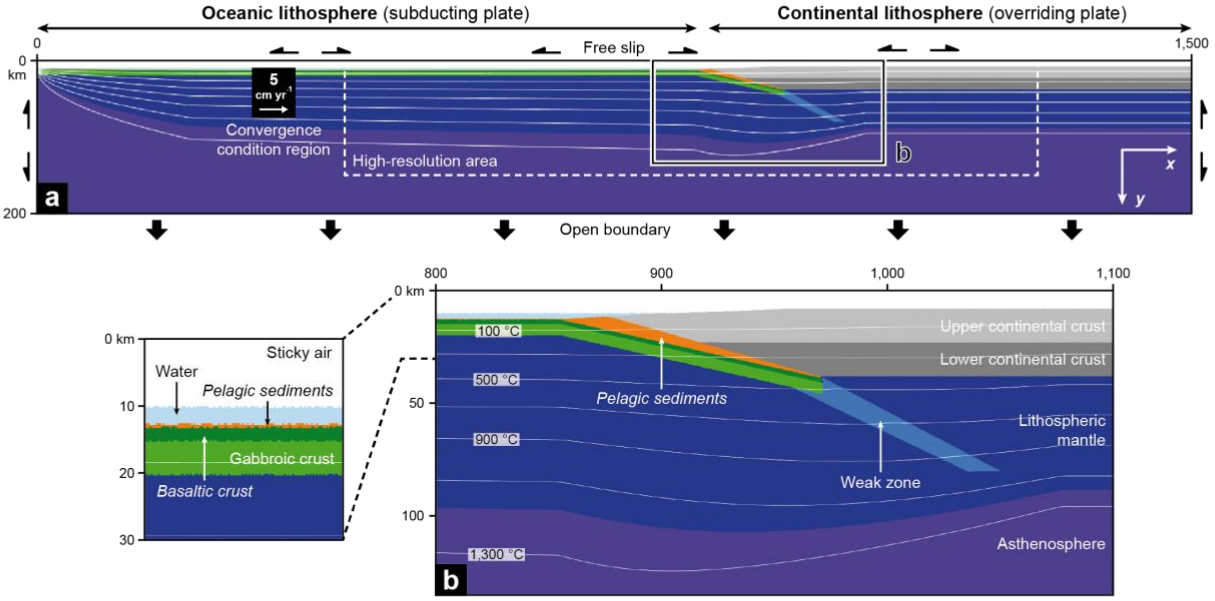

Figure S1. 2-D numerical modelling setup. (**a**) Compositional map of the whole computational domain. (**b**) Zoom of the compositional map on the plate boundary. Rock types with initially-prescribed pore water content are italicized. Details on the layering of the oceanic crust is shown as inset. Note that *y* coordinates on this figure and in *Methods* section are defined from the top of computational domain, while in the main text, model depth is defined as below sea level (i.e., y = 10 km).

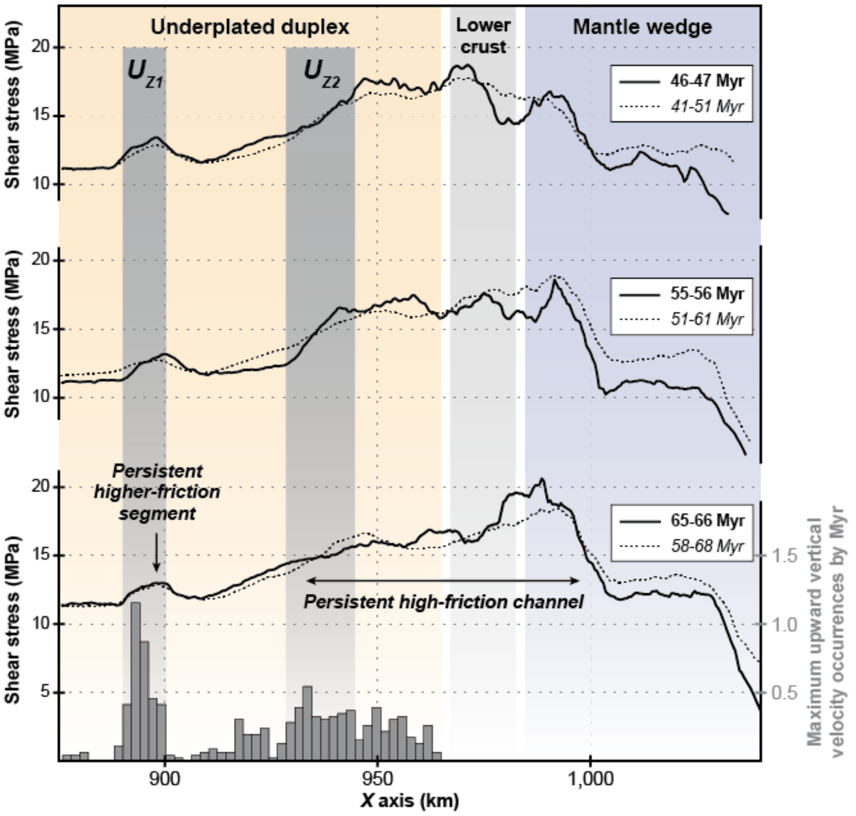

Figure S2. Relations between shear-stress variations along the subduction channel and underplating events in a cold subduction setting (reference model *cold30-5-1*). Line charts show the shear-stress evolution within the subduction channel integrated over 1-Myr-long and 10-Myr-long periods (solid and dotted lines, respectively) for 3 different times of the experiment. Bar chart shows the horizontal distribution of the maximum upward vertical velocity component at the top of the subduction channel for each time step (from 23 to 68 Myr, 479 measurements), which is thought to reflect the distribution of underplating events along the plate interface. The main underplating loci U_{Z1} and U_{Z2} are highlighted by two grey bands. Note the stability of the interface frictional properties all experiment along and the spatial correlation between U_{Z1} and U_{Z2} and the subduction segments exhibiting an increasing frictional behaviour.

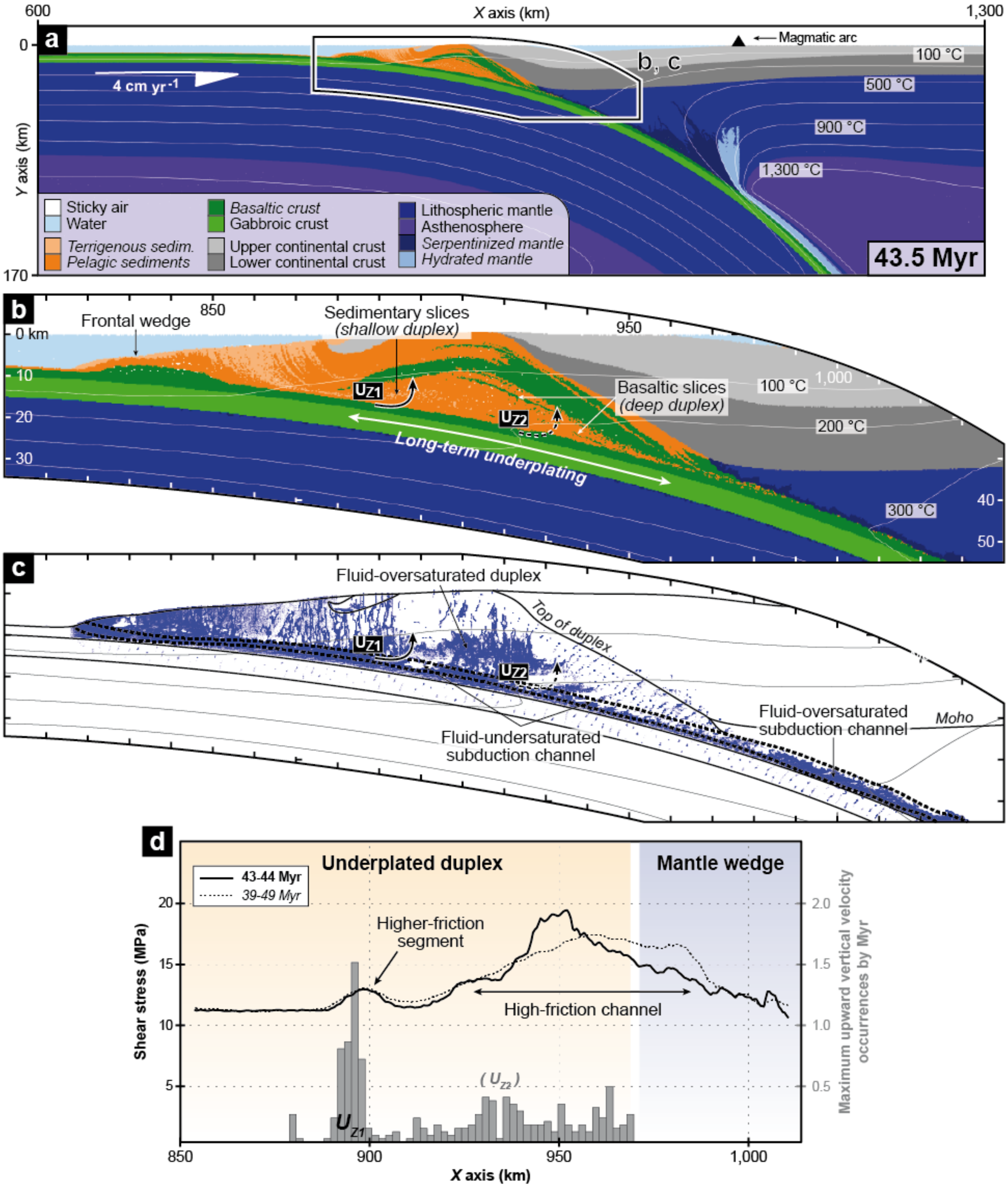

Figure S3. Cold subduction zone with slow plate convergence rate ($v_{conv} = 4$ cm yr^{-1}, model *cold30-4-0.1*). (**a**) Compositional map. Overview of the subduction zone. Rock types with initially-prescribed pore water content are italicized. (**b**) Compositional map. Zoom on the fore-arc region. (**c**) Fluid-distribution map. Fluid markers (in blue) indicate local fluid oversaturation. Preferential loci for underplating U_{Z1} and U_{Z2} are also shown. Thick dashed black lines depict the subduction channel. (**d**) Line chart shows the shear-stress evolution within the subduction channel integrated over 1-Myr-long and 10-Myr-long periods (solid and dotted lines, respectively). Bar chart shows the horizontal distribution of the maximum upward vertical velocity component at the top of the subduction channel for each time step (from 23 to 57 Myr, 366 measurements), which is thought to reflect the distribution of underplating events along the plate interface.

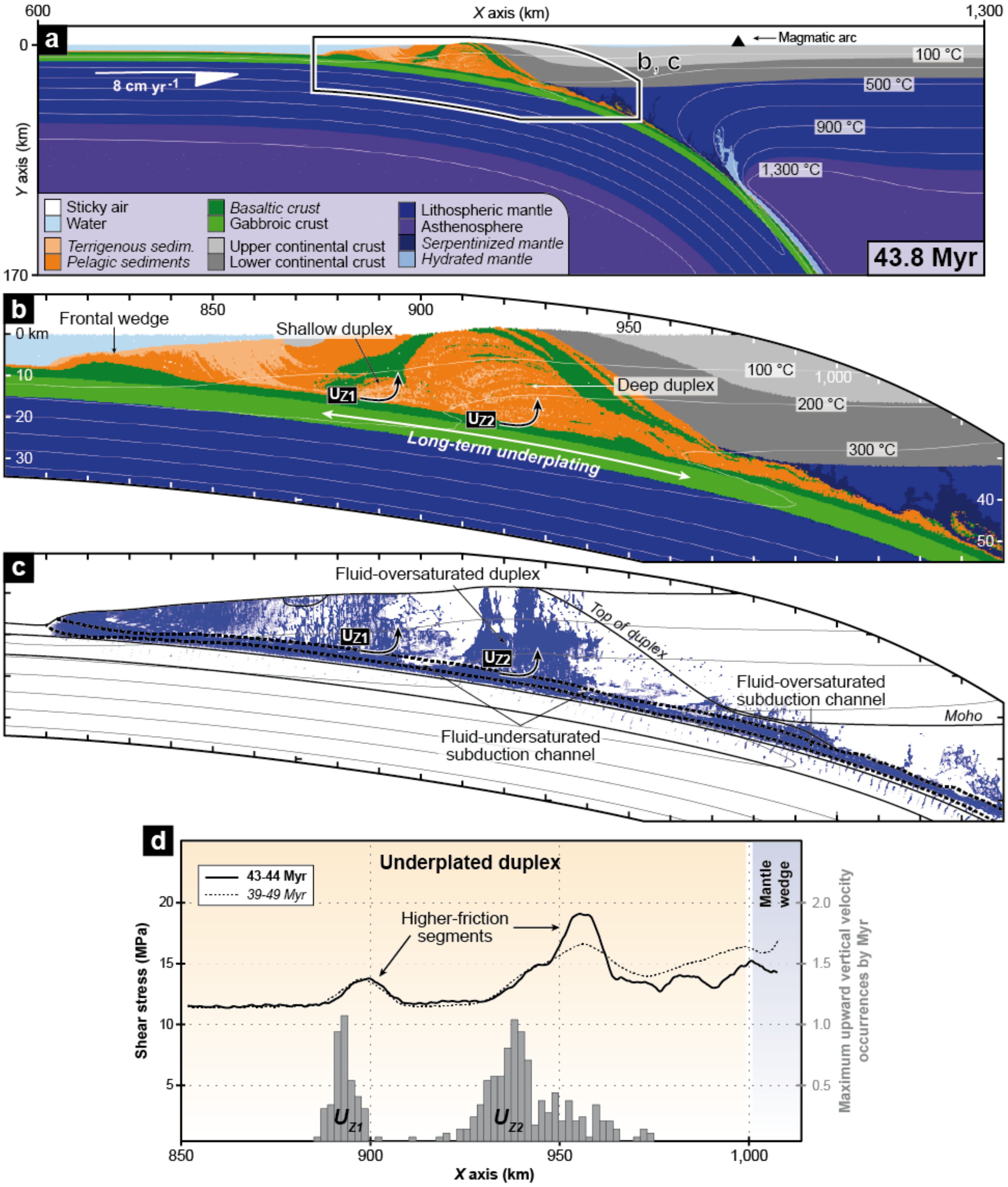

Figure S4. Cold subduction zone with fast plate convergence rate (v_{conv} = 8 cm yr^{-1}, model *cold30-8-0.1*). (**a**) Compositional map. Overview of the subduction zone. Rock types with initially-prescribed pore water content are italicized. (**b**) Compositional map. Zoom on the fore-arc region. (**c**) Fluid-distribution map. Fluid markers (in blue) indicate local fluid oversaturation. Preferential loci for underplating U_{Z1} and U_{Z2} are also shown. Thick dashed black lines depict the subduction channel. (**d**) Line chart shows the shear-stress evolution within the subduction channel integrated over 1-Myr-long and 10-Myr-long periods (solid and dotted lines, respectively). Bar chart shows the horizontal distribution of the maximum upward vertical velocity component at the top of the subduction channel for each time step (from 20 to 49 Myr, 371 measurements), which is thought to reflect the distribution of underplating events along the plate interface.

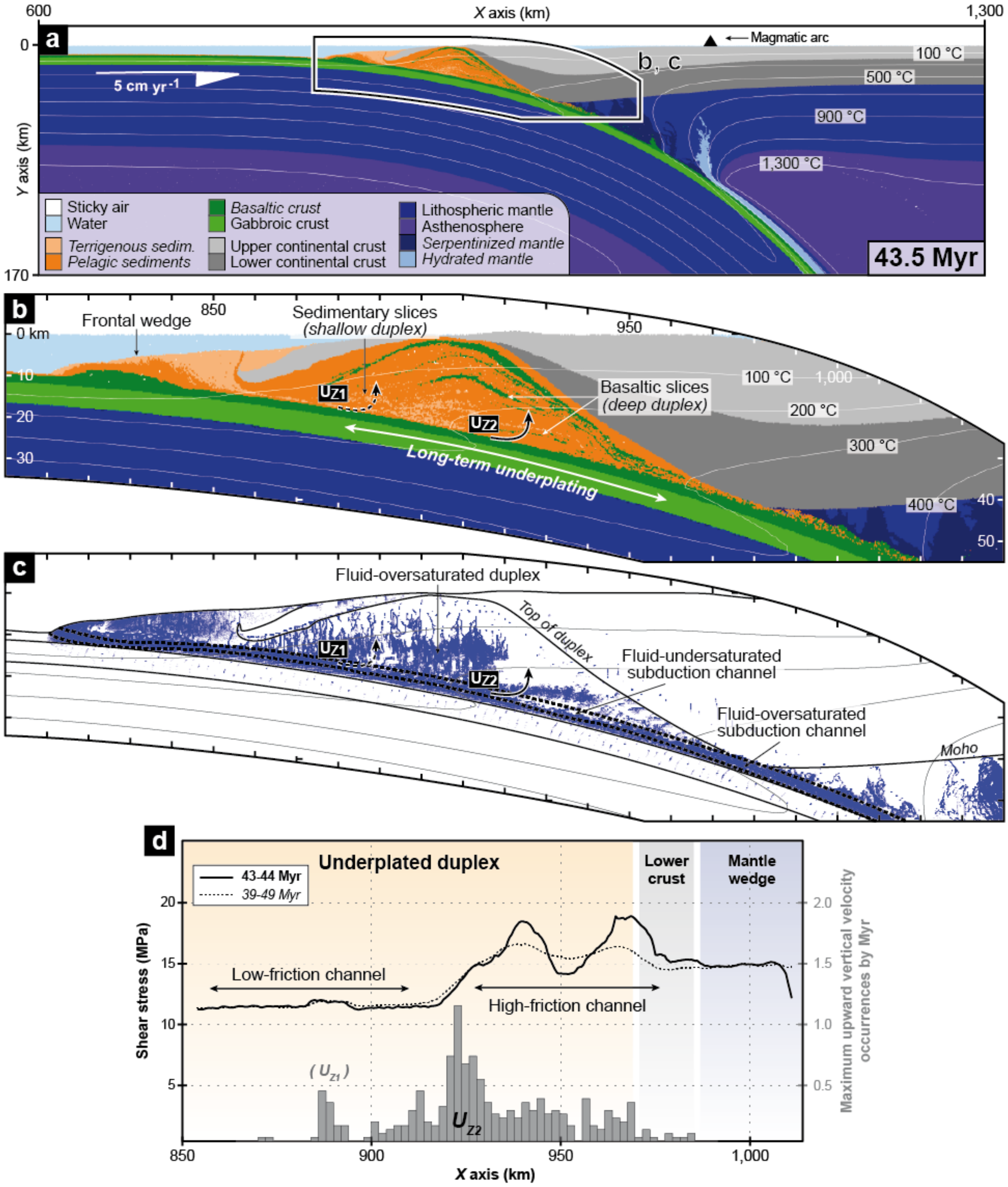

Figure S5. Cold subduction zone with a thick overriding continental crust (i.e., 40 km, model *cold40-5-0.1*). (**a**) Compositional map. Overview of the subduction zone. Rock types with initially-prescribed pore water content are italicized. (**b**) Compositional map. Zoom on the fore-arc region. (**c**) Fluid-distribution map. Fluid markers (in blue) indicate local fluid oversaturation. Preferential loci for underplating U_{Z1} and U_{Z2} are also shown. Thick dashed black lines depict the subduction channel. (**d**) Line chart shows the shear-stress evolution within the subduction channel integrated over 1-Myr-long and 10-Myr-long periods (solid and dotted lines, respectively). Bar chart shows the horizontal distribution of the maximum upward vertical velocity component at the top of the subduction channel for each time step (from 20 to 54 Myr, 357 measurements), which is thought to reflect the distribution of underplating events along the plate interface.

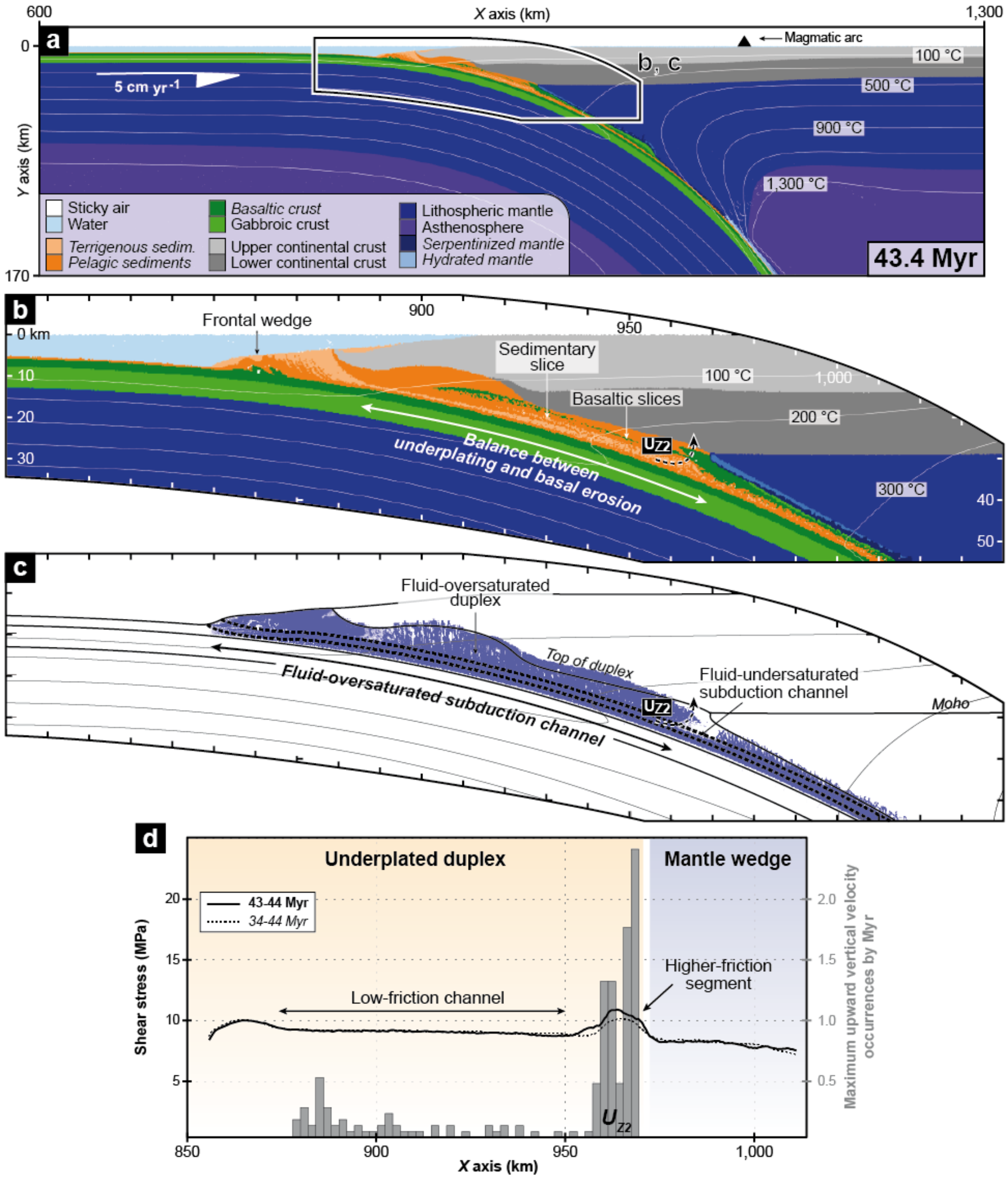

Figure S6. Cold subduction zone with lower reference percolation velocity (v_{perc} = 0.1 cm yr^{-1}, model *cold30-5-0.1*). (**a**) Compositional map. Overview of the subduction zone. Rock types with initially-prescribed pore water content are italicized. (**b**) Compositional map. Zoom on the fore-arc region. (**c**) Fluid-distribution map. Fluid markers (in blue) indicate local fluid oversaturation. Preferential locus for underplating U_{Z2} is also shown. Thick dashed black lines depict the subduction channel. (**d**) Line chart shows the shear-stress evolution within the subduction channel integrated over 1-Myr-long and 10-Myr-long periods (solid and dotted lines, respectively). Bar chart shows the horizontal distribution of the maximum upward vertical velocity component at the top of the subduction channel for each time step (from 23 to 44 Myr, 214 measurements), which is thought to reflect the distribution of underplating events along the plate interface.

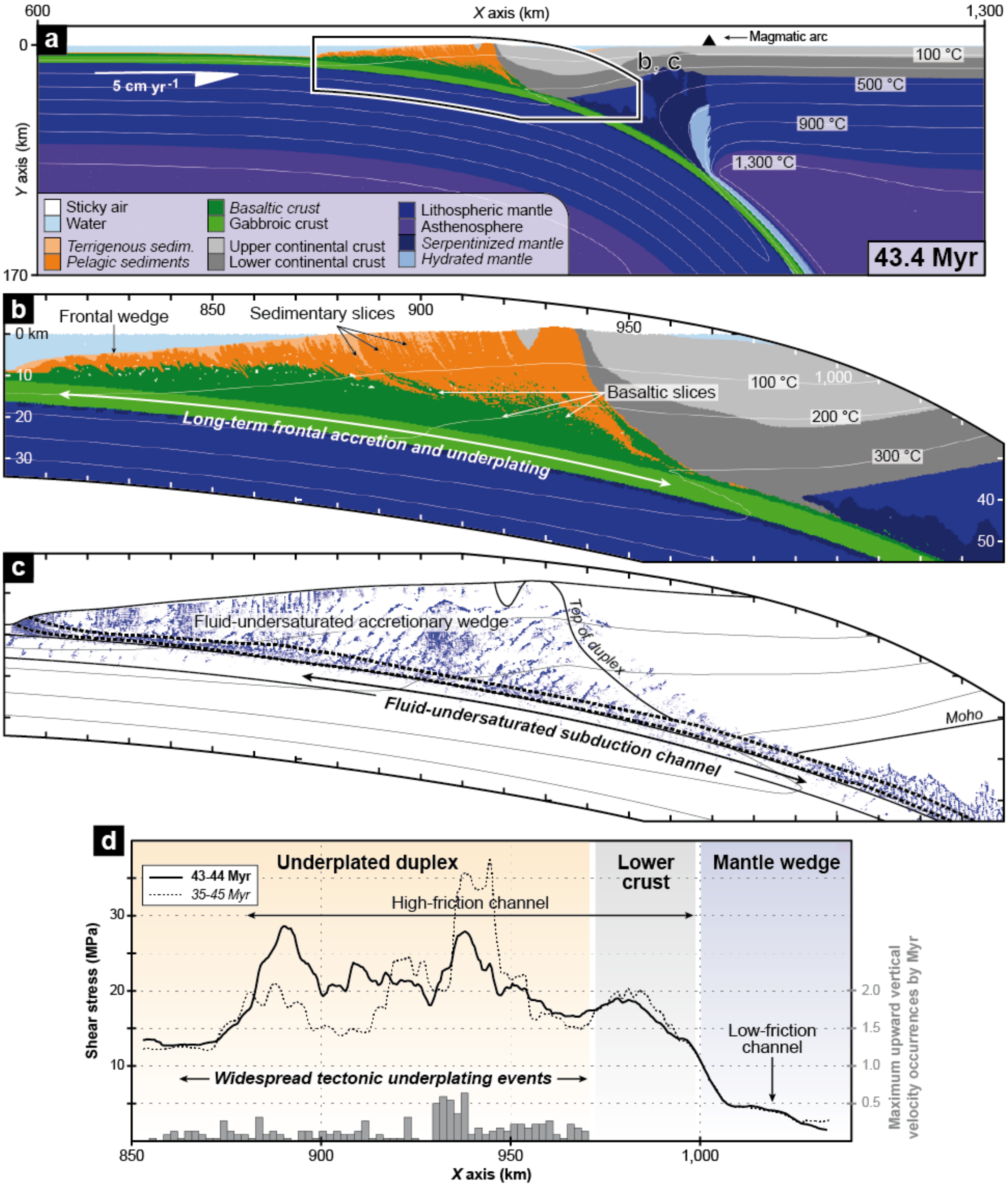

Figure S7. Cold subduction zone with higher reference percolation velocity (v_{perc} = 10 cm yr^{-1}, model *cold30-5-10*). (**a**) Compositional map. Overview of the subduction zone. Rock types with initially-prescribed pore water content are italicized. (**b**) Compositional map. Zoom on the fore-arc region. (**c**) Fluid-distribution map. Fluid markers (in blue) indicate local fluid oversaturation. Thick dashed black lines depict the subduction channel. (**d**) Line chart shows the shear-stress evolution within the subduction channel integrated over 1-Myr-long and 10-Myr-long periods (solid and dotted lines, respectively). Bar chart shows the horizontal distribution of the maximum upward vertical velocity component at the top of the subduction channel for each time step (from 23 to 46 Myr, 230 measurements), which is thought to reflect the distribution of underplating events along the plate interface.

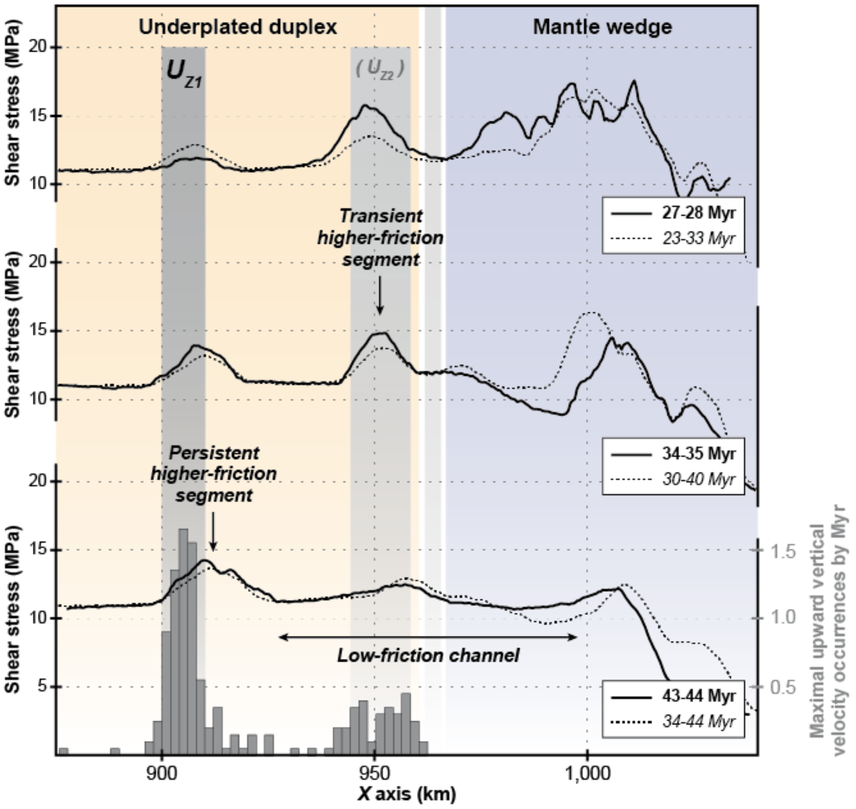

Figure S8. Relations between shear-stress variations along the subduction channel and underplating events in a warm subduction setting (model *warm30-5-1*). Line charts show the shear-stress evolution within the subduction channel integrated over 1-Myr-long and 10-Myr-long periods (solid and dotted lines, respectively) for 3 different times of the experiment. Bar chart shows the horizontal distribution of the maximum upward vertical velocity component at the top of the subduction channel for each time step (from 23 to 44 Myr, 220 measurements), which is thought to reflect the distribution of underplating events along the plate interface. The main underplating loci U_{Z1} and U_{Z2} are highlighted by two grey bands. Note the relative stability of the interface frictional properties all experiment along and the spatial correlation between U_{Z1} and U_{Z2} and the subduction segments exhibiting an increasing frictional behaviour. However, it is noteworthy that the higher-friction segment at x = 950 km disappears after ~35 Myr. As a consequence, tectonic underplating is not active anymore at U_{Z2} after ~35 Myr and basal erosion progressively takes place.

Table S1. Summary of varying model parameters and main results of numerical experiments.

	Varying model parameters				Results		
Model	Oceanic-plate cooling age at the trench (Myr)	Convergence rate (cm yr^{-1})	Overriding-crust thickness (km)	Reference percolation velocity (cm yr^{-1})	Subduction channel	Long-term accretionary dynamics	Figure
cold30-5-1 (reference)	53	5	30	1	Local fluid undersaturation and high-friction patches	Tectonic underplating	Figs. 1, S2
warm30-5-1	**20**	5	30	1	Fluid-oversaturated, low-friction channel	Tectonic underplating and basal erosion	Figs. 4, S8
cold30-4-1	53	**4**	30	1	Local fluid undersaturation and high-friction patches	Tectonic underplating	Fig. S3
cold30-8-1	53	**8**	30	1	Local fluid undersaturation and high-friction patches	Tectonic underplating	Fig. S4
cold40-5-1	53	5	**40**	1	Local fluid undersaturation and high-friction patches	Tectonic underplating	Fig. S5
cold30-5-0.1	53	5	30	**0.1**	Fluid-oversaturated, low-friction channel	Poorly-efficient accretion	Fig. S6
cold30-5-10	53	5	30	**10**	Fluid-undersaturated, high-frictional channel	Dominant frontal accretion	Fig. S7

Table S2. Physical properties of rocks used in numerical experiments. Rock types with initially-prescribed pore water content ($X_{w_{pore}}$ = 1 wt. %) are italicized. Flow laws of all rock types are from ref. 69 except serpentinized mantle, which is from ref. 70.

	Ductile rheology					Plastic rheology		Elastic properties
Material	Flow law	Pre-exponential factor A_D	Creep exponent n	Activation energy E	Activation volume V	Cohesion	Internal friction angle $\sin(\varphi_{dry})$	Shear modulus μ
		(Pa^{-n} s^{-1})		(J mol^{-1})	(J Pa^{-1} mol^{-1})	(Pa)		(Pa)
Sediments (pelagic, terrigenous)	Wet quartzite	1.97×10^{17}	2.3	1.54×10^{5}	8.0×10^{-6}	1.0×10^{7}	0.15	1.0×10^{10}
Upper continental crust	Wet quartzite	1.97×10^{17}	2.3	1.54×10^{5}	1.2×10^{-5}	1.0×10^{7}	0.15	2.5×10^{10}
Lower continental crust	Plagioclase An_{75}	4.80×10^{22}	3.2	2.38×10^{5}	8.0×10^{-6}	1.0×10^{7}	0.15	2.5×10^{10}
Basaltic crust	Plagioclase An_{75}	4.80×10^{22}	3.2	2.38×10^{5}	8.0×10^{-6}	1.0×10^{7}	0.65	2.5×10^{10}
Gabbroic crust	Diabase	1.26×10^{24}	3.4	2.60×10^{5}	8.0×10^{-6}	1.0×10^{7}	0.60	2.5×10^{10}
Dry mantle	Dry olivine	3.98×10^{16}	3.5	5.32×10^{5}	8.0×10^{-6}	1.0×10^{7}	0.60	6.7×10^{10}
Hydrated mantle	Wet olivine	5.01×10^{20}	4.0	4.70×10^{5}	8.0×10^{-6}	1.0×10^{7}	0.10	6.7×10^{10}
Serpentinized mantle	Serpentine	3.21×10^{36}	3.8	8.90×10^{3}	3.2×10^{-8}	1.0×10^{7}	0.10	6.7×10^{10}
Weak zone (hydrated mantle)	Wet olivine	5.01×10^{20}	4.0	4.70×10^{5}	8.0×10^{-6}	1.0×10^{7}	0.10	6.7×10^{10}

Table S2 *(continued)*.

	Density calculation			Heat conservation equation		
Material	Density ρ_0	Thermal expansion α	Compressibility β	Isobaric heat capacity C_P	Thermal conductivity k	Radiogenic heat production H_r
	(kg m^{-3})	(K^{-1})	(Pa^{-1})	(J kg^{-1} K^{-1})	(W m^{-1} K^{-1})	(W kg^{-1})
Sediments (pelagic, terrigenous)	2600	3.0×10^{-5}	1.0×10^{-11}	1.0×10^{3}	[0.64+807/(T+77)] $e^{(4\times P)}$	2.0×10^{-6}
Upper continental crust	2700	3.0×10^{-5}	1.0×10^{-11}	1.0×10^{3}	[0.64+807/(T+77)] $e^{(4\times P)}$	1.0×10^{-6}
Lower continental crust	2950	3.0×10^{-5}	1.0×10^{-11}	1.0×10^{3}	[1.18+474/(T+77)] $e^{(4\times P)}$	1.0×10^{-6}
Basaltic crust	3000	3.0×10^{-5}	1.0×10^{-11}	1.0×10^{3}	[1.18+474/(T+77)] $e^{(4\times P)}$	2.5×10^{-7}
Gabbroic crust	3000	3.0×10^{-5}	1.0×10^{-11}	1.0×10^{3}	[1.18+474/(T+77)] $e^{(4\times P)}$	2.5×10^{-7}
Dry mantle	3200	3.0×10^{-5}	1.0×10^{-11}	1.0×10^{3}	[0.73+1293/(T+77)] $e^{(4\times P)}$	2.2×10^{-8}
Hydrated mantle	3200	3.0×10^{-5}	1.0×10^{-11}	1.0×10^{3}	[0.73+1293/(T+77)] $e^{(4\times P)}$	2.2×10^{-8}
Serpentinized mantle	3000	3.0×10^{-5}	1.0×10^{-11}	1.0×10^{3}	[0.73+1293/(T+77)] $e^{(4\times P)}$	2.2×10^{-8}
Weak zone (hydrated mantle)	3200	3.0×10^{-5}	1.0×10^{-11}	1.0×10^{3}	[0.73+1293/(T+77)] $e^{(4\times P)}$	2.2×10^{-8}

Additional references

66. Clift, P. & Vannucchi, P. Controls on tectonic accretion versus erosion in subduction zones: Implications for the origin and recycling of the continental crust. *Rev Geophys* **42**, RG2001 (2004).
67. Martinod, J. *et al.* How do subduction processes contribute to forearc Andean uplift? Insights from numerical models. *J Geodyn* **96**, 6–18 (2016).
68. Kukowski, N., Lallemand, S. E., Malavieille, J., Gutscher, M.-A. & Reston, T. J. Mechanical decoupling and basal duplex formation observed in sandbox experiments with application to the Western Mediterranean Ridge accretionary complex. *Mar Geol* **186**, 29–42 (2002).
69. Ranalli, G. *Rheology of the Earth*. (1995).
70. Hilairet, N. *et al.* High-Pressure Creep of Serpentine, Interseismic Deformation, and Initiation of Subduction. *Science* **318**, 1910–1913 (2007).
